# Microbial alginate production, modification and its applications

**DOI:** 10.1111/1751-7915.12076

**Published:** 2013-08-19

**Authors:** Iain D Hay, Zahid Ur Rehman, M Fata Moradali, Yajie Wang, Bernd H A Rehm

**Affiliations:** 1 Institute of Fundamental Sciences, Massey UniversityPrivate Bag 11222, Palmerston North, New Zealand; 2 MacDiarmid Institute for Advanced Materials and Nanotechnology, Massey UniversityPalmerston North, New Zealand

## Abstract

Alginate is an important polysaccharide used widely in the food, textile, printing and pharmaceutical industries for its viscosifying, and gelling properties. All commercially produced alginates are isolated from farmed brown seaweeds. These algal alginates suffer from heterogeneity in composition and material properties. Here, we will discuss alginates produced by bacteria; the molecular mechanisms involved in their biosynthesis; and the potential to utilize these bacterially produced or modified alginates for high-value applications where defined material properties are required.

## Introduction

**Figure 1:**
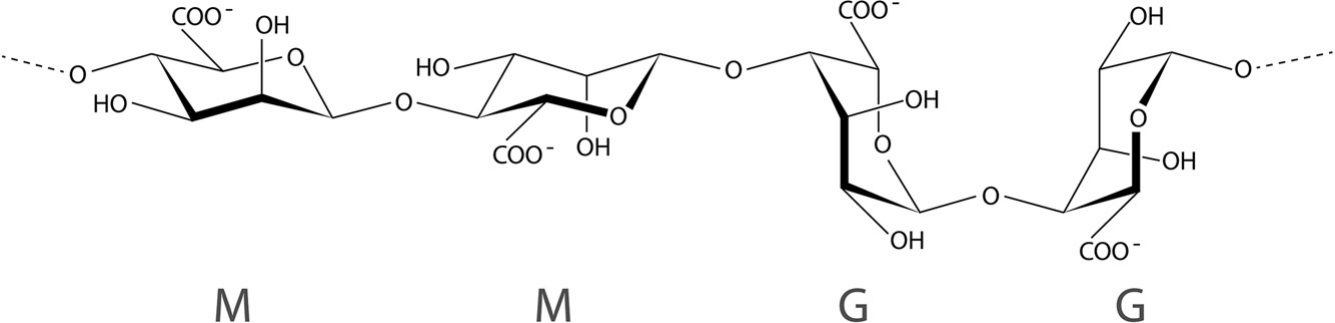
Chemical structure of alginate. M – mannuronate residues, G – guluronate residues.

Alginates are a polysaccharides composed of variable ratios of β-D-mannuronate (M) and its C-5 epimer α-L-guluronate (G) linked by 1–4 glycosidic bonds (Fig. 1). Alginates were first isolated from brown seaweeds in the 1880s, and its commercial production begun in the early 20th century. Alginate can be produced by various genera of brown seaweed and two genera of bacteria, *Pseudomonas* and *Azotobacter*. The ratio of M and G residues and thus the material properties vary depending on the source of the alginate. Its unique physical properties enable it to be used as a stabilizer, viscosifier and gelling agent in the food, beverage, paper, printing and pharmaceutical industries. Industrial production of alginate is estimated to be at least 30,000 metric tons annually with all of that coming from farmed brown seaweed, primarily from the genera *Laminaria* and *Macrocystis*. In these seaweeds, alginate plays a structural role and constitutes up to 40% of the dry matter of the plant; it is thought to play a role analogous to cellulose in terrestrial plants (Draget *et al*., 2005; Donati and Paoletti, 2009). Recently, the desirable material properties as well as its apparent biocompatibility (Klock *et al*., 1997) has led to it being used increasingly in the medical, pharmaceutical and biotechnology industries for applications such as wound dressings (Thomas, 2000); the encapsulation or controlled release of drugs, enzymes or cells; or as a matrices for tissue engineering (Andersen *et al*., 2012; Lee and Mooney, 2012).

## Microbial biosynthesis of alginate

Two genera of bacteria have been shown to secrete alginate, *Pseudomonas* and *Azotobacter*. Most of the research into the molecular mechanisms behind bacterial alginate biosynthesis has been conducted on the opportunistic human pathogen *Pseudomonas aeruginosa* or the soil dwelling *Azotobacter vinelandii*. Although these two genera utilize very similar molecular mechanisms to produce alginate, in nature, they secrete alginate for different purposes with different material properties: Some *P*. *aeruginosa* strains (known as mucoid strains) can secrete copious amounts of alginate to aid in the formation of thick highly structured biofilms (Nivens *et al*., 2001; Hay *et al*., 2009a), whereas *Azotobacter* produces a stiffer alginate (with typically a higher concentrations of G residues) which remains closely associated with the cell and allows the formation of desiccation resistant cysts (Sabra and Ping Zeng, 2009).

The genes involved in alginate biosynthesis are virtually identical between *Pseudomonas* and *Azotobacter* though their regulation is slightly different. All but one of the core genes involved in alginate biosynthesis are contained within a single 12-gene operon initially described by Chitnis and Ohman (1993): *algD*, *alg8*, *alg44*, *algK*, *algE* (*algJ*), *algG*, *algX*, *algL, algI*, *algJ* (*algV*), *algF* and *algA* (*Pseudomonas* gene names are shown with the corresponding *Azotobacter* gene names in parentheses). The genes are under the tight control of a promoter upstream of *algD* (Schurr *et al*., 1993; Shankar *et al*., 1995), although there is some evidence to suggest that alternative internal promoters exist within the operon (Lloret *et al*., 1996; Paletta and Ohman, 2012). The gene *algC* is not located within the operon and is also involved in rhamnolipid and lipopolysaccharide biosynthesis (Goldberg *et al*., 1993; Ye *et al*., 1994; Olvera *et al*., 1999). In addition to these 13 core genes involved in alginate biosynthesis, many more have been identified and are summarized in Table 1. The steps of alginate biosynthesis can be loosely divided into four steps: precursor synthesis, polymerization, periplasmic modification/transit and export.

**Table 1:** Proteins involved in alginate biosynthesis

Protein	Description	Subcellular location	Reference
Core biosynthesis			
AlgA	Precursor synthesis. Phosphomannose isomerase/GDP-mannose pyrophosphorylase.	Cytosol	(Shinabarger *et al*., 1991b)
AlgC	Precursor synthesis. Phosphomannomutase. PDB: 1P5G	Cytosol	(Ye *et al*., 1994)
AlgD	Precursor synthesis. GDP-mannose dehydrogenase. PDB: 1 MV8	Cytosol	(Tatnell *et al*., 1994)
Alg8	Polymerization. Proposed glycosyltransferase/polymerase.	IM	(Remminghorst *et al*., 2009)
Alg44	Polymerization and post transcriptional regulation. c-di-GMP binding and response.	IM	(Remminghorst and Rehm, 2006a)
AlgK	Export/structural role. Lipoprotein, Stabilizes AlgE in OM. PDB: 3EB4	Associated with periplasmic side of OM	(Keiski *et al*., 2010)
AlgE	Export. OM porin. Named AlgJ in *Azotobacter*. PDB: 3RBH	OM	(Whitney *et al*., 2011)
Modification			
AlgG	M-G epimerization. Mannuronan C-5-epimerase	Periplasm	(Franklin *et al*., 1994)
AlgL	Alginate lyase. Control MW, clear alginate from the periplasm.	Periplasm	(Jain and Ohman, 2005)
AlgI	O-Acetylation	IM	(Franklin and Ohman, 2002)
AlgJ	O-Acetylation. Named AlgV in *Azotobacter*	IM	(Franklin and Ohman, 2002)
AlgF	O-Acetylation	Periplasm	(Franklin and Ohman, 2002)
AlgX	O-Acetylation. Structural role. Sequesters MucD. Structural role. PDB: 4KNC	Periplasm	(Gutsche *et al*., 2006; Riley *et al*., 2013)
AlgE1-E7	*Azotobacter* extracellular Mannuronan C-5-epimerases. PDB: 2PYG (AlgE4)	Extracellular	(Ertesvåg *et al*., 2009)
PA1167	Alginate lyase (polyguluronate lyase). PBD: 1VAV	Unknown	(Yamasaki *et al*., 2004)
Regulation			
AlgU (AlgT, σ^22^)	Alternative σ factor homologous to *E. coli* σ^E^ global stress response factor. Positive regulator	Cytosol	(Xie *et al*., 1996)
MucA	Anti σ factor. Negative regulator	IM	(Xie *et al*., 1996)
MucB	Stabilizes MucA. Negative regulator	Periplasm	(Cezairliyan and Sauer, 2009)
MucC	Unclear regulatory role	Periplasm/IM	(Boucher *et al*., 1997a)
MucD	Homologous to *E. coli* serine protease DegP. Negative regulator. Associated with Alginate complex. Negative regulator	Periplasm	(Wood and Ohman, 2006; Hay *et al*., 2012)
AlgW	Homologous to *E. coli* serine protease DegS. Cleaves MucA. Positive regulator	IM	(Cezairliyan and Sauer, 2009)
MucP	Homologous to *E. coli* RseP protease. Positive regulator, cleaves MucA	IM	(Qiu *et al*., 2007)
Prc	Protease. Positive regulator, cleaves MucA	Periplasm	(Wood *et al*., 2006)
MucE	Positive regulator, activates AlgW	OM/Periplasm	(Qiu *et al*., 2007)
ClpX/ClpP/ClpP2	Cytoplasmic proteases. Positive regulators, cleave MucA	Cytoplasm	(Qiu *et al*., 2008)
AlgR	Two-component regulator (Cognate sensor is AlgZ/FimS). Positive regulator, binds to 3 regions in the *algD* promoter	Cytoplasm	(Ma *et al*., 1998)
AlgB	NtrC-Family two-component regulator (Cognate sensor is KinB). Positive regulator, binds to one region in the *algD* promoter	Cytoplasm	(Ma *et al*., 1998)
AmrZ	Arc-like DNA-binding protein. Positive regulator, binds to one region in the *algD* promoter (originally named AlgZ). PDB: 3QOQ	Cytoplasm	(Baynham and Wozniak, 1996; Baynham *et al*., 2006)
AlgQ (AlgR2)	Positive regulator of nucleoside diphosphokinase, necessary for the formation of GDP-mannose	Cytoplasm	(Kim *et al*., 1998)
AlgP (AlgR3)	Histone-like protein required for normal alginate expression, but does not appear to bind *algD* promoter	Cytoplasm	(Kato *et al*., 1990)

### Alginate precursor synthesis

**Figure 2:**
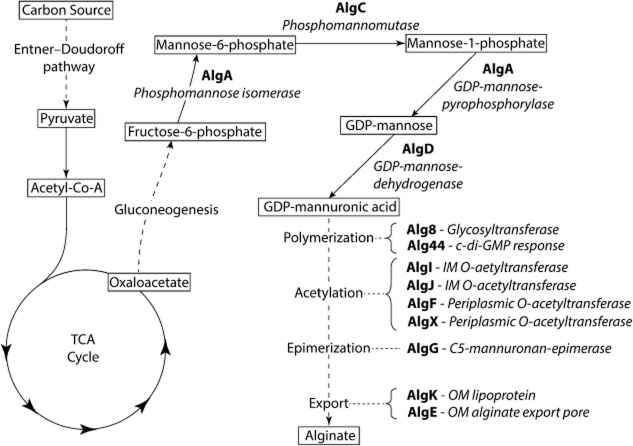
Overview of bacterial alginate biosynthesis.

The formation of the activated precursor guanosine diphosphate (GDP)-mannuronic acid is a well-characterized process and is summarized in Fig. 2. It involves a series of cytosolic enzymatic steps feeding in to the membrane bound alginate polymerization machinery. Synthesis starts with the entry of six carbon substrates into the Entner–Doudoroff pathway, resulting in pyruvate, which is channelled towards the tricarboxylic acid cycle. Subsequently, oxaloacetate is converted to fructose-6-phosphate via gluconeogenesis (Lynn and Sokatch, 1984; Narbad *et al*., 1988). Three well-characterized enzymes, AlgA, AlgC and AlgD, catalyse the four next biosynthesis steps to convert fructose-6-phosphate to GDP-mannuronic acid and these enzymes have been. First, the conversion of fructose-6-phosphate to mannose-6-phosphate is catalysed by the phosphomannose isomerase activity of the bifunctional protein AlgA (May *et al*., 1994). Then, AlgC (phosphomannomutase) converts mannose-6-phosphate to mannose-1-phosphate (Zielinski *et al*., 1991) followed by the conversion to GDP-mannose which is catalysed by the GDP-mannose pyrophorylase activity of AlgA via the hydrolysis of GTP (Shinabarger *et al*., 1991a). Interestingly, this AlgA catalysed step favours the reverse reaction, but the pull of the subsequent AlgD catalysed step shifts the reaction towards GDP-mannose production. The final step is catalysed by AlgD (GDP-mannose dehydrogenase) and is irreversible resulting in GDP-mannuronic acid, which is substrate for the alginate polymerization machinery. The AlgD catalysed oxidation step is thought to be a key rate-limiting reaction in the alginate synthesis pathway (Roychoudhury *et al*., 1989; Tatnell *et al*., 1994; Tavares *et al*., 1999).

### Polymerization

Polymerization and translocation are relatively poorly understood processes in alginate biosynthesis. Disruption mutagenesis shows that at least two proteins are required for polymerization: the inner membrane (IM) proteins Alg8 and Alg44 (Remminghorst and Rehm, 2006a; 2006b). Disruption of the alginate biosynthesis genes generally either results in one of three phenotypes: no loss of alginate production (AlgI, AlgJ and AlgF) (Franklin and Ohman, 2002); loss of alginate production but release alginate fragments due to the activity of a periplasmic alginate lyase (AlgX, AlgG, AlgK and AlgE) (Jain and Ohman, 1998; Jain *et al*., 2003; Gutsche *et al*., 2006; Hay *et al*., 2010); or complete loss of alginate production and no alginate fragments, as is the case for Alg8 and Alg44 (Remminghorst and Rehm, 2006a; 2006b; Oglesby *et al*., 2008).

Bioinformatic analysis suggests that Alg8 is the best candidate for a polymerase. It is predicted to be a glycosyltransferase (family-2 GT), which catalyses the transfer of a sugar molecule from an activated donor to an acceptor molecule (e.g. a growing carbohydrate chain). In accordance with functionally similar transmembrane glycosyltransferases such as AcsAB (cellulose synthase) and Ch1 (chitin synthase), Alg8 showed a predicted structure with several transmembrane domains flanking a long cytoplasmic loop accommodating conserved motifs and catalytic residues. Site-directed mutagenesis of these predicted catalytic residues resulted in loss of alginate biosynthesis. Further experimental support for the direct involvement of Alg8 in alginate polymerization is the observation that overexpression of Alg8 led to overproduction of alginate causing a supermucoid phenotype (Hay *et al*., 2009a). This overproduction would seem to suggest that the polymerization reaction catalysed by Alg8 is a bottleneck in the biosynthesis pathway. Interestingly, in-vitro polymerization experiments showed that the entire cell envelope (IM and outer membrane plus associated proteins) was required for polymerization, suggesting that Alg8 requires other proteins for function (Remminghorst and Rehm, 2006b; Oglesby *et al*., 2008; Remminghorst *et al*., 2009).

The specific role Alg44 plays in polymerization and the mechanisms involved remain unclear, but Alg44 is thought to play an indirect role. Similar to *alg8*, deletion of *alg44* gene resulted in no alginate polymerization, while its overexpression led to overproduction of alginate. Alg44 is predicted as a multidomain protein which consists of a cytoplasmic PilZ domain, a transmembrane region and a periplasmic domain which shows homology to the membrane fusion protein MexA, a membrane-bridging protein involved in the multidrug efflux system of *P. aeruginosa* (Remminghorst and Rehm, 2006a; Oglesby *et al*., 2008). The periplasmic membrane fusion protein domain suggests that Alg44 may play a structural role in bridging the membrane bound polymerase to the periplasmic and outer membrane components facilitating the transit, modification and secretion of alginate. The cytosolic PilZ domain of Alg44 has been shown to bind the bacterial secondary messenger bis-(3′–5′)-cyclic dimeric guanosine monophosphate (c-di-GMP) and is essential for alginate biosynthesis (Merighi *et al*., 2007). Currently, it is unclear how the binding of c-di-GMP by Alg44 is conveyed to the polymerase (Alg8). Similar c-di-GMP-dependent carbohydrate polymerization processes have been observed in various other systems, and these can give us clues as to how this mechanism function here (Weinhouse *et al*., 1997; Franklin *et al*., 2011; Whitney *et al*., 2012; Steiner *et al*., 2013). In one recent study, it was shown that binding of c-di-GMP to the BcsA subunit of the cellulose synthase causes local conformational changes allowing UDP-glucose to access the catalytic site (Morgan *et al*., 2013).

### Periplasmic translocation and modification

After polymerization, the nascent alginate chain (poly-M) is translocated across the periplasm by a putative multiprotein scaffold consisting of at least the periplasmic proteins AlgX, AlgG and AlgK (Jain and Ohman, 1998; Jain *et al*., 2003; Robles-Price *et al*., 2004). These proteins are thought to guide the alginate chain through the periplasm while protecting it from degradation by the periplasmic alginate lyase, AlgL. Intriguingly, it has been proposed AlgL itself also contributes to the formation of a stable periplasmic scaffold (Jain and Ohman, 2005). When components of the periplasmic scaffold are missing, alginate chain leaks into the periplasm where it is degraded by AlgL, releasing free uronic acid oligomers.

Initially, it seems somewhat counterintuitive to find an alginate degrading protein, AlgL, encoded within the alginate biosynthesis operon. Disruption of the *algL* gene in mucoid strains or mutation of the catalytic residues has proved to be difficult, often resulting in non-viability, or loss of mucoidity because of secondary mutations turning off alginate production. This suggests that both AlgL and its lyase activity are required for viability in mucoid strains, presumably serving a maintenance role by degrading misguided alginate trapped in the periplasm. AlgL may also actively control the length of the polymer as well as contribute to periplasmic translocation (Albrecht and Schiller, 2005; Bakkevig *et al*., 2005; Jain and Ohman, 2005). Recently, the details of the reaction catalysed by AlgL from *P. aeruginosa* were characterized. AlgL specifically cleaves the alginate chain via beta elimination, producing mannuronic acids with unsaturated non-reducing ends; the initial steps of this reaction are similar to epimerization. It was found to be a highly processive enzyme that operates preferentially on non-acetylated poly-mannuronan, though it was found to lack strict stereospecificity; it could cleave MM, MG and GG bonds yielding dimeric and trimeric products (Farrell and Tipton, 2012).

Although several studies have indicated that AlgX is essential for alginate production, until recently its exact role remained unclear. Robles-Price and colleagues (2004) proposed that AlgX forms part of the periplasmic scaffold facilitating alginate translocation and secretion. Studies by Gutsche and colleagues (2006) suggested that AlgX was also required for efficient polymerization. The high sequence identity between AlgX and AlgJ, a protein involved in the O-acetylation of alginate, may represent a shared domain for alginate binding. Recently, the structure of AlgX was solved, and it was shown to have two domains: an N terminal SGNH hydrolase domain involved in the acetylation of alginate and a C-terminal carbohydrate-binding module which is thought to aid in alginate binding and orientation (Riley *et al*., 2013).

AlgK, another protein essential for successful translocation of alginate through the periplasm, has an unclear function. AlgK has multiple tetratricopeptide-like (TPR-like) repeats, a feature characteristic of proteins involved in the assembly of multiprotein complexes. This suggests that AlgK may play an important role in the assembly of functional alginate biosynthesis machinery. Keiski and colleagues (2010) recently showed that AlgK is a lipoprotein associated with the outer membrane; moreover, it was shown that AlgK is required for localization of the alginate of the porin AlgE to the outer membrane.

**Figure 3:**
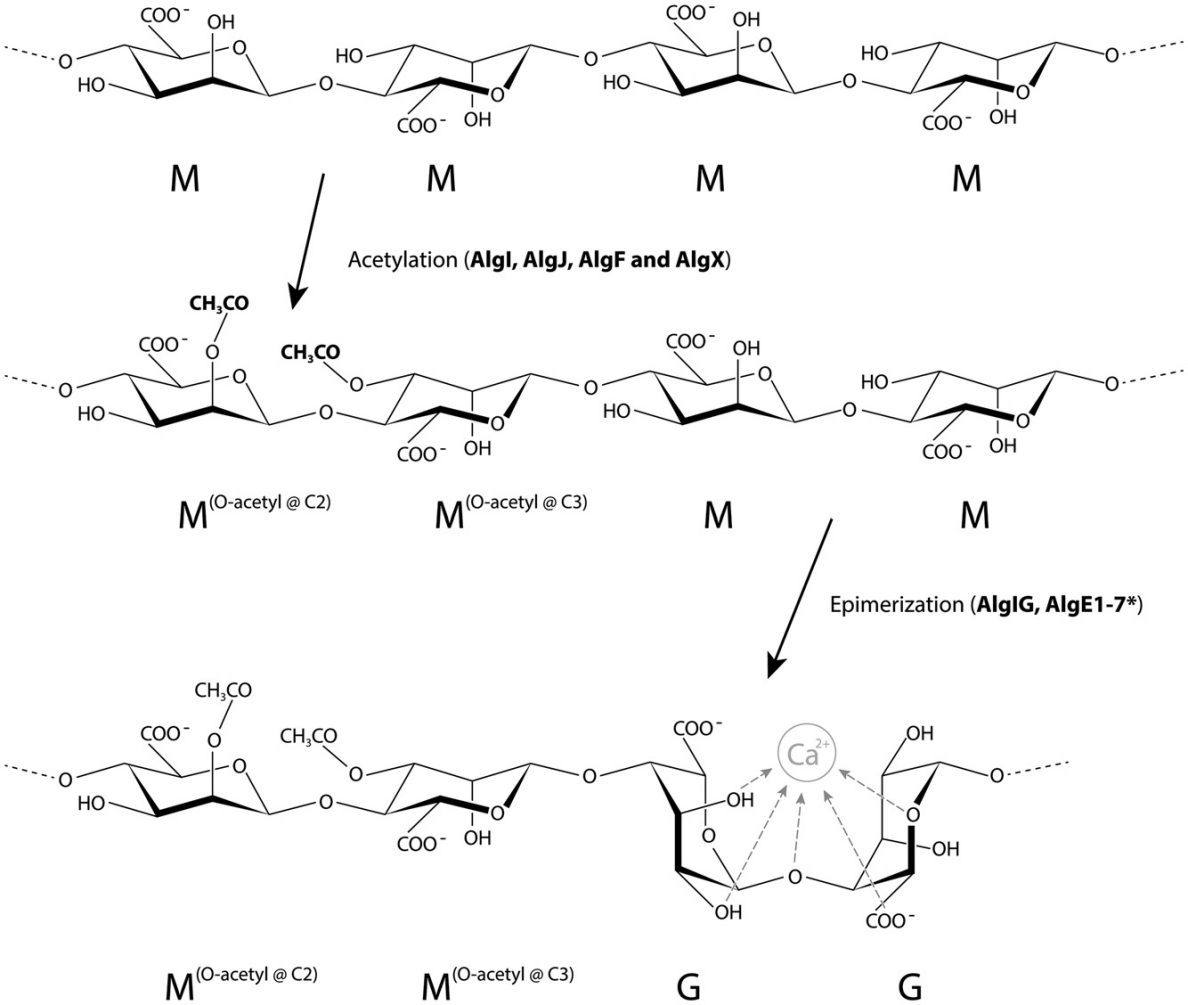
Modification of bacterial alginate. Showing the acetylation of the first two M residues at C2 and C3 respectively; and the C5 epimerization of the third and forth M residues to G residues. The Ca^2+^ binding associated with G-blocks is shown. *AlgE1-7 are extracellular epimerases unique to Azotobacter.

While the nascent alginate chain is transported across the periplasmic space, it can be modified by O-acetylation and epimerization. Although the order of modification remains unknown, it is conceivable that O-acetylation precedes epimerization, as O-acetylation blocks subsequent epimerization or cleavage (Fig. 3). Although neither O-acetylation nor epimerization are essential for alginate production, they can significantly alter the material properties of the resulting alginate (Donati and Paoletti, 2009). The O-acetylation of alginate is unique to bacterial alginates and significantly increased the water holding capacity of alginate; it is required for efficient biofilm development by Pseudomonads as well as protecting the organism from immune responses (Nivens *et al*., 2001; Pier *et al*., 2001).

During its transit through the periplasm, the nascent alginate is O-acetylated by the combined activities of AlgI, AlgJ and AlgF; however, these proteins are not essential for alginate production. AlgX has also recently been implicated in acetylation of alginate; the Ser-His-Asp catalytic triad found in the SGNH-like hydrolase domain was shown to be essential for acetylation (Riley *et al*., 2013). These proteins add O-acetyl ester linkages at the C2 or C3 position of M residues (Fig. 3) (Franklin and Ohman, 2002; Franklin *et al*., 2004). The source of the acetyl group is currently unknown; however, acetyl-coenzyme A is the likely candidate. The acetyl group is thought to be transported to the periplasm by AlgI, a cytoplasmic membrane protein with limited homologies to a *Bacillus subtilis* protein Ipa-4r (DltB), which transports an activated precursor during lipoteichoic acid biosynthesis (Franklin and Ohman, 1996). The second enzyme required for O-acetylation, AlgJ, is a periplasmic protein associated with the cytoplasmic membrane which shows high homology to AlgX; both proteins have sugar-binding-hydrolysing domains likely to be involved in substrate binding (Robles-Price *et al*., 2004). Interestingly, *algI* and *algJ* are thought to be acquired by lateral gene transfer (Franklin *et al*., 2004). AlgF is unique in that it does not have sequence homology to other proteins involved in O-acetylation. Because O-acetylation can restrict epimerization and cleavage, being able to control the level of O-acetylation would allow some level of control over the extent of epimerization and molecular weight.

Epimerization of M residues to G residues leads to changes in material properties. Generally, the presence of G residues in alginates allows for the formation of gels in the presence of divalent cations such as Ca^2+^. The G residues must be found as consecutive stretches (designated ‘G-blocks’) to bind Ca^2+^ (Fig. 3). The overall amount and length of these G blocks affect several properties of the gels, including stiffness, swelling and porosity (Donati and Paoletti, 2009). G-blocks also allow for interchain ion binding in an ‘egg-box'-like structure: the divalent cation interacts with two neighbouring G residues as well as with two G residues in a second chain generating interchain linkages. This is essential for the formation of hydrogels, with higher concentration of G blocks leading to stiffer gels. It is thought that MM or MG blocks are generally incapable of binding divalent cations in this way and are thought to act as elastic ‘hinges’ between the cross linked chains, though Donati and co-workers (2005) have also demonstrated the ability of MG blocks to bind Ca^2+^ and to form gels in a similar manner.

AlgG is responsible for the periplasmic epimerization of M to G residues. AlgG is a bifunctional protein which specifically catalyses the epimerization of M residues to G via protonation-deprotonation of C5 on the M residue in the alginate; it also forms an essential part of the periplasmic scaffold which protects the nascent alginate chain from AlgL-mediated degradation. Epimerization is not essential for the production of high molecular weight (HMW) alginates; mutations disabling the catalytic residues of AlgG do not affect alginate yield (Gimmestad *et al*., 2003; Jain *et al*., 2003).

The catalytic residues of AlgG reside in a shallow groove situated in a right-handed beta-helix fold, a common motif of carbohydrate-binding and sugar-hydrolysing proteins (Douthit *et al*., 2005). The kinetics of this enzyme have been thoroughly examined, demonstrating that AlgG has higher affinity to larger substrates up to 20 residues (100 Å long) suggesting that several AlgG proteins may bind alginate simultaneously (Jerga *et al*., 2006b). While an apparent equilibrium of 75% G content is reached when AlgG is incubated with poly-M substrate *in vitro*, the G content of alginate produced by *P. aeruginosa* is significantly lower (typically less than 40%), suggesting that strict regulation and/or competition between modification pathways is occurring *in vivo* (Schurks *et al*., 2002; Jerga *et al*., 2006a).

It should be noted that in addition to periplasmic AlgG, *A. vinelandii* also possess at least seven extracellular alginate epimerases, AlgE1-E7 with differing specificities and non-random epimerization patterns (Ertesvåg *et al*., 2009).

### Alginate secretion

The outer membrane beta barrel porin, AlgE, is responsible for the secretion of mature alginate (Hay *et al*., 2010). This protein is immunogenic and displays anion selectivity upon spontaneous incorporation into planar lipid bilayers (Rehm *et al*., 1994a; 1994b). Recently, the crystal structure of AlgE has been determined (Whitney *et al*., 2011) and functional residues of the protein thoroughly probed (Rehman and Rehm, 2013). Despite the lack of sequence similarity, AlgE was found to be structurally similar to OprD, a substrate-specific nutrient uptake channel. The AlgE pore is lined with highly conserved, charged amino acid residues, in-part formed by the extracellular loops L3 and L7 folding into the lumen of the pore, which have been suggested to confer selectivity towards alginate and/or facilitate its efficient secretion across the outer membrane. AlgE has an unusually long and flexible periplasmic loop (L8) which appears to act as a ‘stopper’ in the deduced structure. It is thought that this region may interact with other subunits of the alginate biosynthesis machinery such as the TPR domains of AlgK and/or the membrane fusion domains of Alg44. It has been proposed that AlgK and AlgE interact, and this pair shares homology to enzymes involved in cellulose, Pel and poly-β-1,6-N-acetyl-D-glucosamine biosynthesis (Keiski *et al*., 2010; Rehm, 2010).

### Multiprotein alginate polymerization/secretion complex

**Figure 4:**
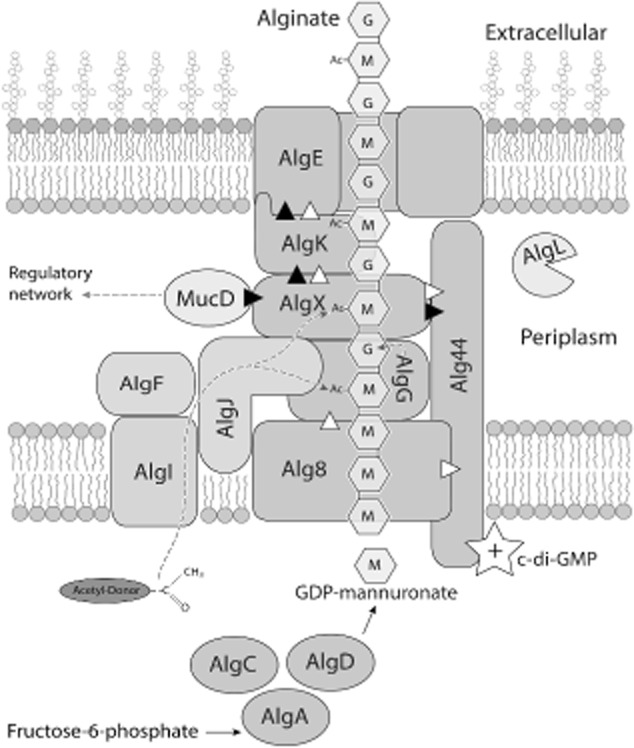
Schematic representation of the alginate polymerization/secretion complex spanning from the inner membrane to the outer membrane. Acetylation by AlgI, AlgJ, AlgF and AlgX and epimerization by AlgG are demonstrated by dashed lines. It remains unclear whether AlgJ or AlgX or both are responsible for the direct acetylation of the alginate chain. Deduced interactions are shown as triangles, with white triangles indicating a mutual stability relationship and black triangles representing a direct interaction as indicated by pull down data.

As mentioned above, it has long been suggested that the members of the alginate biosynthesis machinery form a multiprotein complex spanning from the IM, through the periplasmic space and into the outer membrane. Several studies have elucidated the specific protein–protein interactions involved in this complex (Gutsche *et al*., 2006; Keiski *et al*., 2010; Hay *et al*., 2012; Rehman and Rehm, 2013; Rehman *et al*., 2013). Recently, Rehman and colleagues (2013) have undertaken a series of pull-down, cross-linking and mutual-stability experiments in an effort to map the specific protein–protein interactions in the multiprotein complex. This has led to a model for the alginate polymerization/secretion complex as depicted in Fig. 4 (with experimentally deduced interactions indicated by triangles). Intriguingly, a key regulatory protein, MucD, appears to interact with the complex through AlgX. It is unclear what the function of this interaction is, but it has been suggested that MucD may be sequestered by an intact complex and becomes free to exert its regulatory role if the complex becomes instable (Gutsche *et al*., 2006; Hay *et al*., 2012).

### Regulation

The regulation of alginate biosynthesis is complex and involves transcriptional and post-translational levels of regulation, as well as several hypermutable regions of the genome in which mutant alleles lead to overproduction of alginate. Globally acting regulators as well as several alginate-specific regulators govern alginate biosynthesis. Transcriptional regulation of alginate biosynthesis in *P. aeruginosa* can be loosely divided into two different types: environmental stimuli-based regulation and a ‘genotypic switch'-based form of regulation (Rehm and Valla, 1997).

**Figure 5:**
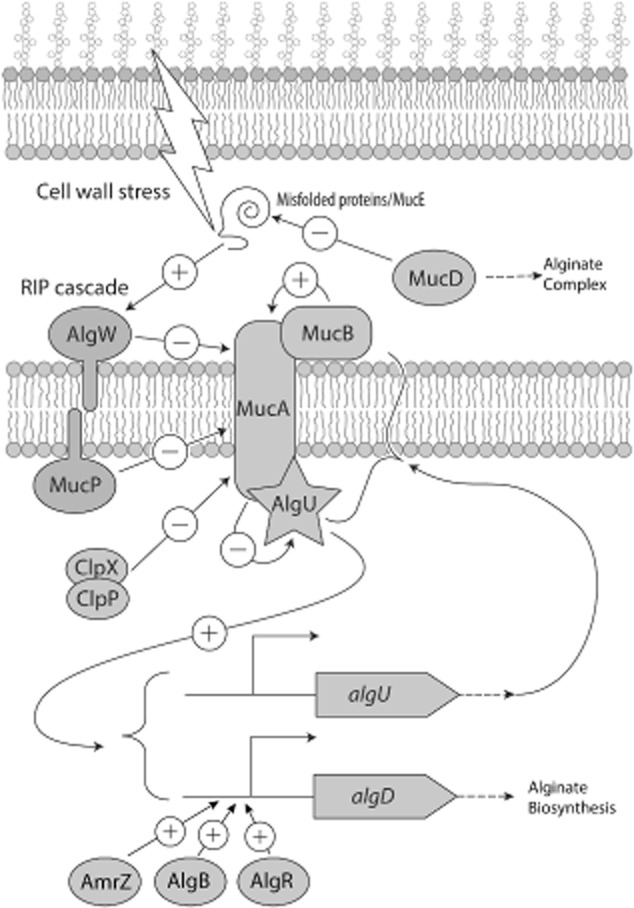
Overview of the regulation of alginate biosynthesis. The periplasmic regulation and the regulated intramembrane proteolysis cascade are shown. The core alginate biosynthesis operon is indicated by the *algD* arrow. The ‘genetic switch’ operon is indicated by the *algU* arrow.

Most of the genes involved in the genotypic switch are located within a single self-regulated operon (*algU, mucA, mucB, mucC* and *mucD*). This region is somewhat homologous to the well-characterized σ^E^ region in *Escherichia coli*, containing the genes *rpoE* (encoding the σ^E^), *rseA, rseB, rseC*. AlgU is a key alternate σ^22^ factor, which is at the apex of a hierarchy of regulators involved in alginate biosynthesis and is ultimately required for transcription starting from the *AlgD* promoter (Chitnis and Ohman, 1993; Deretic *et al*., 1994; Firoved and Deretic, 2003; Ramsey and Wozniak, 2005). AlgU is sequestered at the IM (and thus unable to bind RNA polymerase and initiate transcription) by the membrane anchored anti-sigma factor MucA (Schurr *et al*., 1996; Xie *et al*., 1996; Mathee *et al*., 1997). The periplasmic protein MucB binds to the periplasmic side of MucA and plays a negative regulatory role in alginate biosynthesis by protecting MucA from proteolysis. Release of AlgU and subsequent transcription appears to be initiated by a regulated intramembrane proteolytic (RIP) cascade leading to the degradation of MucA (Wood *et al*., 2006; Cezairliyan and Sauer, 2009). Several steps of the RIP cascade have recently come to light: The periplasmic protease AlgW (*E. coli* DegS homologue) initially cleaves MucA in response to envelope stress. Particular misfolded proteins (in particular, the outer membrane protein MucE) can bind to the PDZ activating domain of AlgW and cause its activation (de-repression). After cleavage by AlgW, MucA becomes susceptible to cleavage on the cytosolic side by the intramembrane protease MucP (*E. coli* RseP/YaeL homologue) leading to the release of AlgU (Qiu *et al*., 2007, Damron and Yu, 2011) (Fig. 5). Three further cytosolic proteases, ClpX, ClpP1 and ClpP2, have recently been shown to be involved in the proteolysis of MucA (Qiu *et al*., 2008). MucD is a periplasmic protease that appears to be playing a role antagonistic to that of AlgW. Disruption of *mucD* gene leads to a mucoid phenotype signifying a negative regulatory role. It is thought that MucD is involved in the degradation of misfolded proteins that would otherwise activate AlgW or MucP (Wood and Ohman, 2006; 2009; Qiu *et al*., 2007; Damron and Yu, 2011). Although alginate production is the most apparent phenotype controlled by AlgU, it does not act exclusively on the alginate operon and has been shown to be involved in the transcriptional activation of genes with diverse functions, including genes involved in biosynthesis of other exopolysaccharides (Firoved *et al*., 2002; Firoved and Deretic, 2003; Ghafoor *et al*., 2011).

This operon has become known as the ‘switch’ locus because of the relatively high number of mutations found in this region in clinical mucoid isolates. The most common mutations occur in MucA and/or MucB, with up to 80% of mucoid *P. aeruginosa* clinical isolates containing mutations in the *mucA* gene. Most of these mutations result in a premature stop codon and a truncated MucA rendering the RIP cascade redundant (Martin *et al*., 1993a; 1993b; Boucher *et al*., 1997b; Ciofu *et al*., 2008; Pulcrano *et al*., 2012).

In addition to AlgU, several other proteins are required to initiate transcription of the alginate operon. This layer of regulation is known as ‘environmental stimuli'-based regulation. AlgR is a response regulator part of a two-component regulator that binds to three sites in the *algD* promoter; the cognate sensory component of this regulatory pair is AlgZ (FimS) and strangely is not required for transcription of the alginate operon. AlgB is also part of a two-component regulator and binds to one site on the *algD* promoter; again, it's activity is apparently independent of its cognate sensor kinase KinB (Ma *et al*., 1998). AmrZ (originally called AlgZ), an Arc-like DNA-binding protein, binds to one site on the *algD* promoter (Baynham and Wozniak, 1996; Baynham *et al*., 1999; 2006).

As mentioned above, the essential alginate biosynthesis protein Alg44 contains a c-di-GMP-binding/sensing PilZ domain in its C-terminus. This allows for an additional post-translational level of regulation (Merighi *et al*., 2007). C-di-GMP is an important bacterial secondary messenger that has been linked to the post-translational regulation of diverse processes such as motility, exopolysaccharide production and virulence (Amikam and Galperin, 2006). Recently, it has been demonstrated that one particular c-di-GMP-synthesizing protein, MucR, specifically influences the levels of alginate biosynthesis in *P. aeruginosa* presumably by generating a localized c-di-GMP pool in proximity to the alginate polymerization/secretion multiprotein complex (Hay *et al*., 2009b).

## Applications of bacterial alginates

All current commercial alginates are isolated from farmed brown seaweeds, with over 30,000 metric tons produced annually (Draget *et al*., 2005). Its material properties, versatility and biocompatibility has led to alginates use as a viscosity regulator and stabilizer in foods, cosmetics and high-value medical applications including wound dressings, drug delivery systems and more recently in tissue encapsulation for regenerative therapy (Tonnesen and Karlsen, 2002; Qin, 2008; Lim *et al*., 2010).

While bulk alginate extracted from seaweed for the food and cosmetic industries is sold at prices as low as $5 kg^−1^, pharmaceutical grade alginates with defined MW, M/G ratios and hence more defined material properties cost more than $100 g^−1^ (Pronova web catalogue prices as of April 2013). The high-value applications of alginate in biotechnology and biomedical sciences require a steady supply of alginates with defined homogeneity in composition and material properties. Although seaweed alginates are extensively used in biomedical applications as an immobilization material, it suffers from problems with mechanical stability, wide pore size distribution and osmotic swelling during physiological conditions. Furthermore, it is subject to heterogeneity in both ratio of G to M residues as well as molecular weight because of environmental and seasonal variation (Draget *et al*., 2005; Mørch *et al*., 2007; Andersen *et al*., 2012). This inability of algal alginate to fulfil specific demands created by biomedical industry can be overcome either by using bacterial alginate or using bacterial enzymes to modify algal alginates (Rehm, 2010).

Because of the pathogenic nature of *P. aeruginosa*, any commercial bacterial production of alginate is likely to come from *A. vinelandii* or non-pathogenic *Pseudomonas* species. The M/G residue composition of *A. vinelandii* alginate is similar to those produced by seaweeds. By exploiting regulatory proteins discussed above, it is possible to engineer *A. vinelandii* strains with increased levels of transcription from the *algD* operon and thus increased levels of alginate production. Indeed, when this was combined with disruption of the polyhydroxybutyrate pathway (thus allowing more carbon sources for alginate biosynthesis) up to 7 g l^−1^ of alginate was obtained (Pena *et al*., 2002; Galindo *et al*., 2007). Furthermore, disruption of the Na^+^-translocating NADH: ubiquinone oxidoreductase complex in *A. vinelandii* leads to an alginate overproducing phenotype, this alginate also had a higher degree of acetylation and a lower G/M ratio, though the exact mechanism remains unclear (Nunez *et al*., 2009; Gaytan *et al*., 2012).

Viscosity is also influenced by the molecular weight of alginate. HMW alginate (more viscous) can be produced by *A. vinelandii* when the dissolved oxygen is controlled and/or the *algL* gene is disrupted (Pena *et al*., 2000; Trujillo-Roldan *et al*., 2003; 2004; Diaz-Barrera *et al*., 2010). Recently, a study has linked the increased expression of alginate polymerase *alg8/alg44* with the production of HMW alginate in *A. vinelandii* (Diaz-Barrera *et al*., 2012). Interestingly, a mutant of *A. vinelandii*, with increased expression of the *algD* operon and disruption of polyhydroxybutyrate biosynthesis, produced alginate with an extremely HMW (4000 kDa) (Pena *et al*., 2002).

As mentioned above, *A. vinelandii* secretes seven C-5 epimerases each introducing a specific ratio and pattern of G residues. For example, AlgE2 and AlgE6 introduces continuous stretches of G-residues forming G-blocks while AlgE4 actions results in the formation of MG blocks (Ertesvåg *et al*., 2009). Utilizing these enzymes allows a tighter control of the material properties of alginate. Indeed, *A. vinelandii* epimerases have been employed to modify alginate to exhibit material properties required for immobilization of living cells (Martinsen *et al*., 1989; Mørch *et al*., 2007). A mutant of *P. fluorescence* lacking the epimerase AlgG can be used to produce poly-M (Gimmestad *et al*., 2003). High M content alginates are of particular practical interest for some types of cell transplantations because of their particular material properties and biocompatibility (Klock *et al*., 1994; 1997); poly-Ms hydrolysis products are known to exert anti-inflammatory activity (Mirshafiey and Rehm, 2009).

As mentioned above, a key difference between algal and bacterial alginates is that the latter is O-acetylated. A higher degree of O-acetylation significantly increases viscosity and pseudoplastic rheology (Skjåk-Bræk *et al*., 1989; Donati and Paoletti, 2009). The level of O-acetylation can be controlled reasonably well using specific strains/mutants or altering the growth media and controlling cultivation conditions such as aeration, pH and temperature (Pena *et al*., 2006; Diaz-Barrera *et al*., 2010; Gaytan *et al*., 2012). In addition, live immobilized *P. syringae* cells have been successfully used to acetylate seaweed-derived alginates (Lee and Day, 1995).

Understanding and harnessing these mechanisms of alginate production and modification in bacteria could enable manufacture of tailor made bacterial alginates for high value medical and biotechnological applications.
